# Complement C3 and Activated Fragment C3a Are Involved in Complement Activation and Anti-Bacterial Immunity

**DOI:** 10.3389/fimmu.2022.813173

**Published:** 2022-02-25

**Authors:** Meng Wu, Bei-bei Jia, Mo-fei Li

**Affiliations:** ^1^ Chinese Academy of Sciences (CAS) & Shandong Province Key Laboratory of Experimental Marine Biology, Institute of Oceanology, Center for Ocean Mega-Science, Chinese Academy of Sciences, Qingdao, China; ^2^ Laboratory for Marine Biology and Biotechnology, Pilot National Laboratory for Marine Science and Technology (Qingdao), Qingdao, China; ^3^ College of Earth and Planetary Sciences, University of Chinese Academy of Sciences, Beijing, China

**Keywords:** C3, C3a, complement, Japanese flounder, bacterial infection

## Abstract

In the complement system, C3 is a central component in complement activation, immune defense and immune regulation. In all pathways of complement activation, the pivotal step is conversion of the component C3 to C3b and C3a, which is responsible to eliminate the pathogen and opsonization. In this study, we examined the immunological properties of C3 and its activated fragment C3a from Japanese flounder (*Paralichthys olivaceus*) (PoC3 and PoC3a), a teleost species with important economic value. PoC3 is composed of 1655 amino acid residues, contains the six domains and highly conserved GCGEQ sequence of the C3 family. We found that *PoC3* expression occurred in nine different tissues and was upregulated by bacterial challenge. In serum, PoC3 was able to bind to a broad-spectrum of bacteria, and purified native PoC3 could directly kill specific pathogen. When PoC3 expression in Japanese flounder was knocked down by siRNA, serum complement activity was significantly decreased, and bacterial replication in fish tissues was significantly increased. Recombinant PoC3a (rPoC3a) exhibited apparent binding capacities to bacteria and Japanese flounder peripheral blood leukocytes (PBL) and induce chemotaxis of PBL. Japanese flounder administered rPoC3a exhibited enhanced resistance against bacterial infection. Taken together, these results indicate that PoC3 is likely a key factor of complement activation, and PoC3 and PoC3a are required for optimal defense against bacterial infection in teleost.

## 1 Introduction

The complement system is composed of approximately 35 proteins, which present in serum, tissue fluid and the surface of cell membranes (1, 2). The complement system plays an important role in host defense, which is widely involved in innate and adaptive immunity (3). The complement system can be activated by three different pathways, including the classical pathway, lectin pathway, and alternative pathway (1, 4, 5). C3 is a center factor in the complement system, all three complement activation pathways converge in C3 and formation of C5 convertase (6). C5 convertase cleavage of C5 to C5a and C5b, and C5b is involved in assembly of the membrane-attack complexes (MAC) (7, 8). MAC forms channels or pores on the cell surface of pathogens, leading to the destruction of infected cells and the death of foreign pathogens (6–8).

In mammals, the molecule weight of C3 is about 190 kDa, and C3 is composed of two polypeptide chains α and β connected by disulfide bonds, and contains a highly conserved GCGEQ motif in α chain (9, 10). The mutation or deletion of the C3 gene leads to a variety of immune diseases, and increases bacterial infections (11). Previous studies have shown that the C3-deficient mice lacked of antibody response to an exogenous antigen and showed decreased immune function (12, 13). In human, hereditary C3 deficiency can cause severe recurrent infections and immune complex disorders (14).

In the cascade reaction of complement activation, C3 convertase cleaves C3 to produce C3a and C3b (6). In mammals, C3a is also known as anaphylatoxin, and it is derived from the N-terminal region of C3 α chain (15). C3a consists of 74-78 amino acids and molecule weight is approximately 10 kDa (15). Previous studies indicated that C3a can bind to specific cell surface receptors and induce chemotaxis and phagocytosis to eliminate pathogen (16–19). In human, recombinant or synthetic C3a can induce the chemotaxis of eosinophils and mast cells, and indirectly activate neutrophils (20–22). In mammals, C3a induces multiple immune responses, including stimulating respiratory burst (20, 21, 23), regulating tumor necrosis factor-α and interleukin 1β expression (22), and killing bacteria and fungi (24, 25).

In teleost, C3 has been cloned and identified in several fish species, including olive flounder (26), southern catfish (27), spotted wolfish (28), rainbow trout (29), Atlantic salmon (30), zebrafish (31), and orange-spotted grouper (32). In teleost, expression pattern of C3 was determined in various tissues with or without pathogen infection, and C3 transcripts was significantly impacted by environment stress (pH and temperature) in tissues (27, 33). In rainbow trout and gilthead sea bream, mature C3 has been purified from serum (34, 35), however, the function of C3 is less known in fish. In *Branchiostoma japonicum*, recombinant C3a can stimulate chemotaxis of macrophages, and enhance phagocytosis and respiratory burst of macrophage (36). In rainbow trout, C3a is also reported to enhance phagocytosis of head kidney leukocytes (37). In this study, with an aim to gain more insights into the function of teleost C3 and C3a, we examined the biological role of Japanese flounder (*Paralichthys olivaceus*) C3 (PoC3) and C3a (PoC3a) in complement activation and antibacterial immune defense.

## 2 Materials And Methods

### 2.1 Ethics Statement

All experiments involving live animals conducted in this study were approved by the Ethics Committee of Institute of Oceanology, Chinese Academy of Sciences. All methods were carried out in accordance with the relevant guidelines, including any relevant details.

### 2.2 Fish

Clinically healthy Japanese flounders (average 24.3 g) were purchased from a local fish farm (Haiyang, Qingdao, China). The fish were acclimatized in the laboratory for 2 weeks at 20°C in aerated seawater prior to the study. Before the experiment, fish were randomly sampled and verified to be absent of bacterial pathogens as reported previously (38). For tissue collection, fish were euthanized as reported previously (39).

### 2.3 Bacterial Strains and Culture Conditions

*Bacillus subtilis, Micrococcus luteus, Pseudomonas fluorescens* TSS, *Vibrio anguillarum* C312, *Edwardsiella tarda* TX1, *Vibrio harveyi* T4D, and *Streptococcus iniae* SF1 are pathogenic bacteria preserved in the laboratory (40–42). *Escherichia coli* DH5α and BL21 (DE3) were purchased from TransGen (Beijing, China). *V. anguillarum, V. harveyi, E. tarda, P. fluorescens*, and *B. subtilis* were inoculated into Luria-Bertani (LB) broth and grew overnight at 28°C. *E. coli* was inoculated into LB and grew overnight at 37°C. *S. iniae* were inoculated to tryptic soy broth (TSB) and grew overnight at 28°C.

### 2.4 Sequence Analysis

BLAST program was used to analyse the amino acid sequence of PoC3 (GenBank accession no. BAA88901.1) at the National Center for Biotechnology Information (NCBI). Domain search, theoretical molecular mass and pI, multiple sequence alignment, and phylogenetic analysis were performed as described previously (38).

### 2.5 Real-Time Quantitative PCR

#### 2.5.1 PoC3 Expression in Fish Tissues Under Normal Physiological Conditions

Under normal condition, different tissues (intestine, head kidney, blood, liver, spleen, heart, gill, muscle, and brain) were taken aseptically from Japanese flounder (5 fish) as above. Total RNA extraction and cDNA synthesis of each tissue were performed as described previously (38, 43). The qRT-PCR was carried out with primers F1 (5’-GGCTCAACTCAGGCTACCATT-3’) and R1 (5’-GAGGTAGAGCATAATACAGCGACA-3’). The expression level of PoC3 was analyzed using comparative threshold cycle method (2^−ΔΔCT^) with β-actin (ACTB) as an internal reference. All data were given in terms of mRNA levels relative to that of ACTB and expressed as means plus or minus standard errors of the means (SEM).

#### 2.5.2 PoC3 Expression During Bacterial Infection

*E. tarda* and *V. anguillarum* are common fish pathogens, and these bacteria were cultured and resuspended in PBS to 10^7^ CFU/ml as described previously (38). Japanese flounders were randomly divided into three groups (20/group) and injected intramuscularly with 100 μl *E. tarda*, *V. anguillarum* or PBS. At 6, 12, 24, and 48 h post-infection, blood, spleen and head kidney were collected from 5 fish, and qRT-PCR was performed to detect the expression of PoC3 as above. The α-tubulin (TUBA) gene was used as an internal reference gene in blood and spleen, and 18S rRNA was used as an internal reference gene in head kidney as reported previously (44).

### 2.6 Binding Assay of PoC3 to Bacteria in Serum

Purification of PoC3 and preparation of mouse anti-PoC3 polyclonal antibody were described previously (45, 46). *V. harveyi*, *E. tarda*, *E. coli*, *S. iniae*, *V. anguillarum* and *P. fluorescens* were grown to mid-log phase as described above. The bacterial cells were harvested by centrifugation and washed with PBS. The bacteria were adjusted to 1×10^9^ CFU/mL in PBS. Serum was added to the bacterial suspension and incubated at room temperature for 1 h. For the control sample, PBS was added to the bacterial suspension. After incubation, the bacteria were centrifuged and washed with PBS for 3 times. The collected bacteria were lysed with 8 M urea, centrifuged at 10,000 rpm for 2 min, and the supernatant was taken for Western blot analysis (45). The interaction between serum PoC3 and bacteria was detected by ELISA as reported previously (47). Bacteria were cultured and resuspended in coating buffer (15 mM Na_2_CO_3_, 35 mM NaHCO_3_, pH 9.6) to 10^8^ CFU/ml as above, and were incubated in the 96-well ELISA plates (Corning Incorporated, New York, USA) at 4°C for overnight. After the sealed with 5% skim milk powder (Solarbio, Beijing, China), the plates were incubated with different dilutions fold of Japanese flounder serum (1/2, 1/4, 1/8, and 1/16) or PBS (control) at 22°C for 2 h, and washed with PBST. Mouse anti-PoC3 polyclonal antibody were added to the respective plates. The plates were incubated at 37°C for 1 h and washed three times in PBST. Goat anti-mouse IgG-horseradish peroxidase (HRP) antibody (Abcam, Cambridge, UK) was added to the plates, and the plates were incubated at 37°C for 1 h. Subsequent ELISA assay was determined as reported previously (47).

### 2.7 Bacterial Killing Assay

*V. harveyi*, *E. tarda*, *E. coli*, *S. iniae*, *V. anguillarum* and *P. fluorescens* were cultured as above, harvested by centrifugation and resuspended in PBS to 10^6^ CFU/ml as above. Purified native PoC3 or bovine albumin (BSA) was added to the bacterial suspension at the final concentration of 12.5 µg/ml, 25 µg/ml, 50 µg/ml, or 100 µg/ml. For the control sample, PBS was added to the bacterial suspension. The mixture was incubated at room temperature for 1 h and then plated on LB or TSB agar plates as described previously (46). The plates were incubated at 28°C for 24 h, and the colonies that appeared on the plates were counted.

### 2.8 PoC3 Knockdown

PoC3 knockdown was performed by small RNA (siRNA) interference as reported previously (47). Briefly, to select PoC3 specific siRNA, three different siRNA targeting PoC3 were inserted into the siRNA expression vector pRNAT-CMV3.1 (GenScript, Piscataway, USA) at BamHI/AlfII sites, resulting in plasmids pPoC3si-1, pPoC3si-2, and pPoC3si-3. In addition, the plasmid pPoC3siC, which expresses a scramble siRNA, was constructed in the same fashion. To examine the efficiency of the siRNA plasmids, four groups (N = 5) of Japanese flounder (average 20.3 g) were injected intramuscularly with each of the plasmids (20 μg/fish) or with PBS. At 7 d post-plasmid administration, expression of PoC3 in blood, kidney, and spleen was determined by qRT-PCR as described above. The plasmid with the strongest inhibitory effect on PoC3 expression was renamed pPoC3si. This screening experiment was performed three times. The siRNA sequences expressed by pPoC3si and pPoC3siC are 5’-CGAACAGTATGAGTGTGTTC-3’ and 5’-CAGTAGGTAACGAACCTGAC-3’ respectively.

### 2.9 Serum Hemolytic and Bactericidal Activity

Serum was prepared from Japanese flounder containing pPoC3si, pPoC3siC or PBS (control) and was diluted serially in Hank’s Balanced Salt Solution (HBSS) (Solarbio, Beijing, China). Hemolytic activity and bactericidal activity assay were determined as reported previously (45, 48). For hemolytic activity, the control sample at 1/4 serum dilution was defined as 100% for easy of comparison. For bactericidal activity, the control sample at 1/8 serum dilution was defined as 100% for easy of comparison.

### 2.10 Effect of PoC3 Knockdown on Bacterial Infection

*E. tarda* was cultured as above and resuspended in PBS to 1×10^8^ CFU/ml. Japanese flounder were divided randomly into three groups (N = 15) and administered with pPoC3si, pPoC3siC or PBS (control) as above. At 7 d post-plasmid administration, the fish were infected with 100 μl *E. tarda* as described above. At 12 h, 24 h, and 48 h post-infection, blood, spleen, and kidney were taken from the fish (5 fish/time point) and examined for bacterial recovery by plate count. The experiment was performed three times.

### 2.11 Preparation of Recombinant PoC3a (rPoC3a)

The coding sequence of *PoC3a* was cloned by PCR with primers F2 (5′-GGATCCATG GCTACCACTGTAATGAACGTC-3′, underlined sequence, BamH1 site) and R2 (5′-CTCGAGTCACTTGTCATCATCGTCTTTGTAATCGCGAGCCAAGTCGAGCTGAT-3′, underlined sequence, Xho1 site and Flag-tag). The PCR product of PoC3a was fused a Flag-tag at the C-terminal. PCR product was ligated with the T-A cloning vector T-Simple (TransGen Biotech, Beijing, China), and the recombinant plasmid was digested with BamH1 and Xho1 to retrieve the PoC3a-containing fragment, which was inserted into pET28a-Sumo that contains 6× His tag and SUMO (small ubiquitin-like modifier) tag at the N-terminal as described previously (49, 50) between BamH1/Xho1 sites, resulting in pEtPoC3a-Sumo. *E. coli* BL21 (DE3) was transformed with pEtPoC3a-Sumo (which expresses the rPoC3a-Sumo) and pET28a-Sumo (which expresses the rSumo). Protein purification from the transformants was performed as reported previously (51). Briefly, the transformants were cultured in LB medium at 37°C to mid-log phase. Isopropyl-β-D-thiogalactopyranoside was added to the culture to a final concentration of 0.5 mM. After growing at 16°C for an additional 16 h, the cells were harvested by centrifugation, and recombinant proteins were purified using nickel-nitrilotriacetic acid columns (GE Healthcare, Piscataway, USA), as recommended by the manufacturer. The purified proteins were dialyzed for 24 h against PBS and treated with Triton X-114 to remove endotoxin as reported previously (52). The reconstituted proteins were treated with Sumo Protease (Thermo, Waltham, America) to remove His- and Sumo-tag. The proteins were analyzed by SDS-PAGE and visualized after staining with Coomassie brilliant blue R-250.

### 2.12 Binding Assay of rPoC3a

#### 2.12.1 Binding to Bacteria

The interaction between protein and bacteria was detected by ELISA as reported previously (47). Eight bacteria were cultured and resuspended in coating buffer (15 mM Na_2_CO_3_, 35 mM NaHCO_3_, pH 9.6) to 10^8^ CFU/ml as above, and were incubated in the 96-well ELISA plates (Corning Incorporated, New York, USA) at 4°C for overnight. After the sealed with 5% skim milk powder (Solarbio, Beijing, China), the plates were coated with different concentrations (0.3125 μg/ml, 0.625 μg/ml, 1.25 μg/ml, 2.5 μg/ml, 5 μg/ml, and 10 μg/ml) of rPoC3a, rSumo (10 μg/ml) or PBS (control) at 22°C for 2 h, and washed with PBST. Mouse anti-Flag antibody targeting Flag-tagged rPoC3a or mouse anti-His antibody targeting His-tagged rSumo (Abcam, Cambridge, UK) were added to the respective plates. The plates were incubated at 37°C for 1 h and washed three times in PBST. Goat anti-mouse IgG-horseradish peroxidase (HRP) antibody (Abcam, Cambridge, UK) was added to the plates, and the plates were incubated at 37°C for 1 h. Subsequent ELISA assay was determined as reported previously (47).

#### 2.12.2 Binding to Peripheral Blood Leukocytes (PBL)

PBL were prepared from Japanese flounder with Percoll and PBL-protein binding was determined by microscopy as reported previously (53). Briefly, PBL were resuspended in PBS to 10^7^ cells/ml and added to glass slide (Citotest, Jiangsu, China) to allow the cells to settle. After 1 h, the supernatant was removed, and the slide was blocked with 5% skim milk powder in PBS and incubated at 22°C for 1 h. The slide was washed with PBS, and rPoC3a (50μg/ml), rSumo (50μg/ml) and PBS (control) were added to the slide. The slide was incubated at 22°C for 1 h and washed with PBS for three times. Mouse anti-Flag antibody targeting Flag-tagged rPoC3a or mouse anti-His antibody targeting His-tagged rSumo was added to the slide. The slide was incubated at 37°C for 2 h and washed as above. Fluorescein isothiocyanate (FITC)-labeled goat antimouse IgG (Abcam, Cambridge, UK) was added to the slide. The slide was incubated at 37°C for 1 h in dark and washed as above. The cells were fixed with 4% paraformaldehyde for 0.5 h and washed as above. The cells were stained with 4, 6-diamino-2-phenylindole (DAPI) (Solarbio, Beijing, China) and examined with a confocal microscope (Carl Zeiss, Oberkochen, Germany).

### 2.13 Chemotaxis Assay

PBL were prepared as above. Chemotaxis assay was carried out in 24-well Costar Transwell (Corning Costar Co., Cambridge, MA, USA) as described previously (54). Briefly, different concentrations (6.25 μg/ml, 12.5 μg/ml, 25 μg/ml and 50 μg/ml) of rPoC3a and rSumo were diluted in L-15 medium. As a control, PBS was similarly diluted. Six hundred microliters of each of the dilutions was applied to the lower chamber of Transwell. The upper chamber containing a poly-carbonate membrane of 3 μm pore size was placed on top of the lower chamber. One hundred microliters of PBL (10^6^ cells/ml) were added to the upper chamber, and the plate was incubated at 22°C for 40 min. The number of cells migrated into the lower chamber was counted with a microscope. Chemotactic index was presented as fold increase in the number of migrated cells induced by purified recombinant protein compared to that induced by PBS. To distinguish between chemotaxis and chemokinesis, the above assay was also performed with the same concentration of rPoC3a present in both the upper and lower chambers of Transwell. The assay was performed independently for three times.

### 2.14 Effect of rPoC3a on Bacterial Infection

Japanese flounder were divided randomly into three groups (N = 15) and rPoC3a, rSumo or PBS (control) were injected intraperitoneally at the dose of 20 μg/fish. *E. tarda* was cultured as above and resuspended in PBS to 10 ^6^ CFU/ml. At 6 h post-protein administration, the fish were infected with 100 μl *E. tarda* as described above. At 12 h, 24 h, and 48 h post-infection, blood, spleen and kidney were taken from the fish (5 fish/time point) and examined for bacterial recovery by plate count. The experiment was performed three times.

### 2.15 Statistical Analysis

All experiments were performed three times, and statistical analyses were carried out with SPSS 18.0 software (SPSS Inc., Chicago, IL, USA). Data were analyzed with analysis of variance (ANOVA), and statistical significance was defined as P < 0.05.

## 3 Results

### 3.1 PoC3 Sequence Analysis

**Figure 1:**
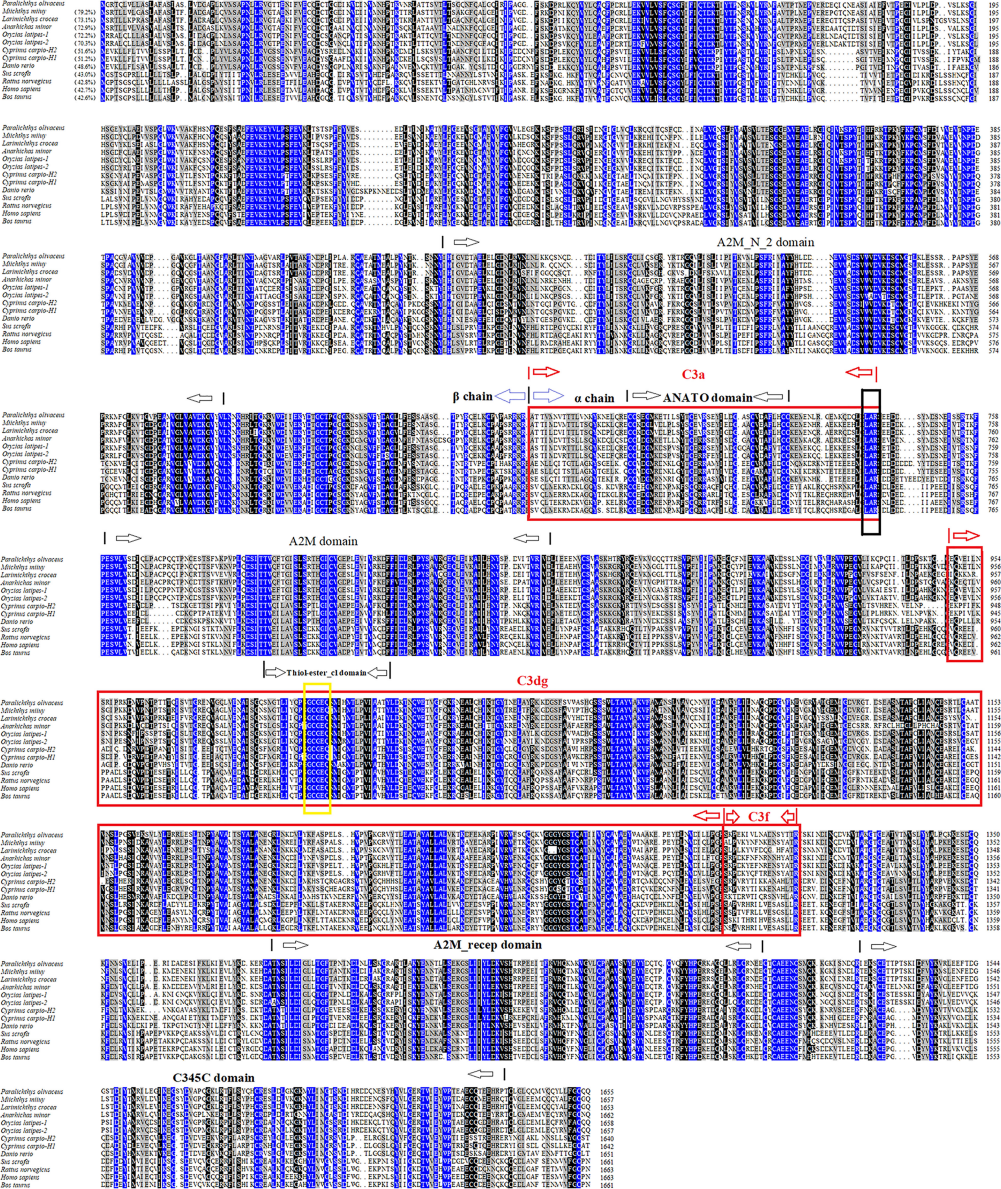
Sequence alignment of PoC3 homologues. Dots denote gaps introduced for maximum matching. Numbers in brackets indicate overall sequence identities between PoC3 and the compared sequences. The consensus residues are in blue, the residues that are ≥75% identical among the aligned sequences are in black, and the residues that are ≥50% identical among the aligned sequences are in grey. The A2M_N_2, ANATO, A2M, Thiol-ester_cl, A2M_recep and C345C domains are marked with black arrows. Derived C3 activation fragment C3a, C3dg, and C3f are marked with red box. The C3 convertase cleavage site of C3 is marked in black box. The highly conserved sequence GCGEQ of the C3 family is marked with a yellow box. The GenBank accession numbers of the aligned sequences are as follows: PoC3, BAA88901.1; *Miichthys miiuy*, AFC89899.1; *Larimichthys crocea*, AHZ41228.1; *Anarhichas minor*, CAC29154.1; *Oryzias latipes*-1, NP_001098552.1; *Oryzias latipes*-2, NP_001098553.1; *Cyprinus carpio-*H2, BAA36620.1; *Cyprinus carpio-*H1, BAA36619.1; *Danio rerio*, NP_001032313.2; *Sus scrofa*, NP_999174.1; *Rattus norvegicus*, NP_058690.2; *Homo sapiens*, NP_000055.2; *Bos taurus*, NP_001035559.2.

**Figure 2:**
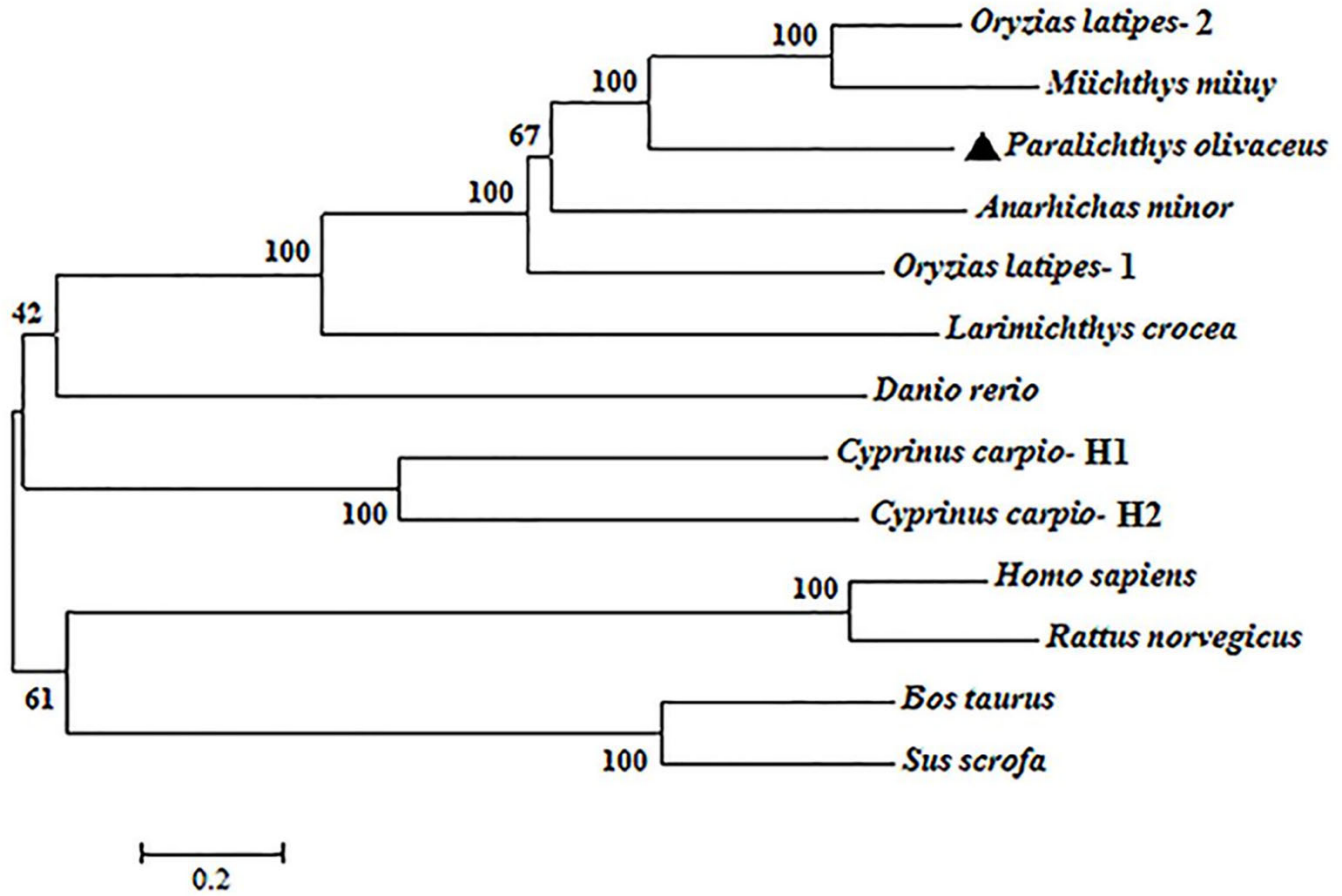
Phylogenetic analysis of PoC3. The phylogenetic tree was constructed using the neighbor-joining method of MEGA-X based on the amino acid sequences from teleost and mammals. Data were analyzed using p-distance, with gaps removed by pairwise deletion. The topological stability of the tree was evaluated by 1000 bootstrap replications. The GenBank accession numbers of the phylogenetic sequences are as follows: *Oryzias latipes*-2, NP_001098553.1; *Miichthys miiuy*, AFC89899.1; *Paralichthys olivaceus*, BAA88901.1; *Anarhichas minor*, CAC29154.1; *Oryzias latipes*-1, NP_001098552.1; *Larimichthys crocea*, AHZ41228.1; *Danio rerio*, NP_001032313.2; *Cyprinus carpio*-H1, BAA36619.1; *Cyprinus carpio-*H2, BAA36620.1; *Homo sapiens*, NP_000055.2; *Rattus norvegicus*, NP_058690.2; *Bos taurus*, NP_001035559.2; *Sus scrofa*, NP_999174.1.

PoC3 is composed of 1655 residues, which has a calculated molecular mass of 184.7 kDa and a theoretical pI of 6.05. PoC3 is composed of α chain (residues 663 to 1653) and ß chain (residues 1 to 662), and possesses 6 domains, A2M_N_2 (α2-macroglobulin family N-terminal region) domain (residues 457 to 597), Anato (anaphylatoxin-like domain) domain (residues 685 to 720), A2M (α2-macroglobulin family domain) domain (residues 760 to 860), and Thiol-ester_cl (α2-macroglobulin thiol-ester bond forming region) domain (residues 992 to 1021), A2M_recep (α2-macroglobulin receptor) domain (residues 1385 to 1479), and C345C (netrin C-terminal domain) domain (residues 1518 to 1637) (**Figure 1**). The predicted derivative C3 activation fragment includes C3a, C3dg, and C3f (**Figure 1**). PoC3 contains the highly conserved sequence GCGEQ (residues 1002 to 1006) of the C3 family. DNAMAN analysis showed that PoC3 shares 42.6% to 79.2% overall sequence identities with the C3 of *Miichthys miiuy*, *Larimichthys crocea*, *Anarhichas minor*, *Oryzias latipes*-1, *Oryzias latipes*-2, *Cyprinus carpio-*H2, *Cyprinus carpio-*H1, *Danio rerio*, *Sus scrofa*, *Rattus norvegicus*, *Homo sapiens*, and *Bos Taurus* (**Figure 1**). Phylogenetic analysis showed that the constructed evolutionary tree was mainly divided into two branches, among which PoC3 was mainly clustered together with teleost C3, including *Oryzias latipes*, *Miichthys miiuy*, *Anarhichas minor*, *Larimichthys crocea*, *Danio rerio* and *Cyprinus carpio*, and it is farther from the branch of mammals (**Figure 2**).

### 3.2 Expression of PoC3 in Absence and Presence of Bacterial Infections

**Figure 3:**
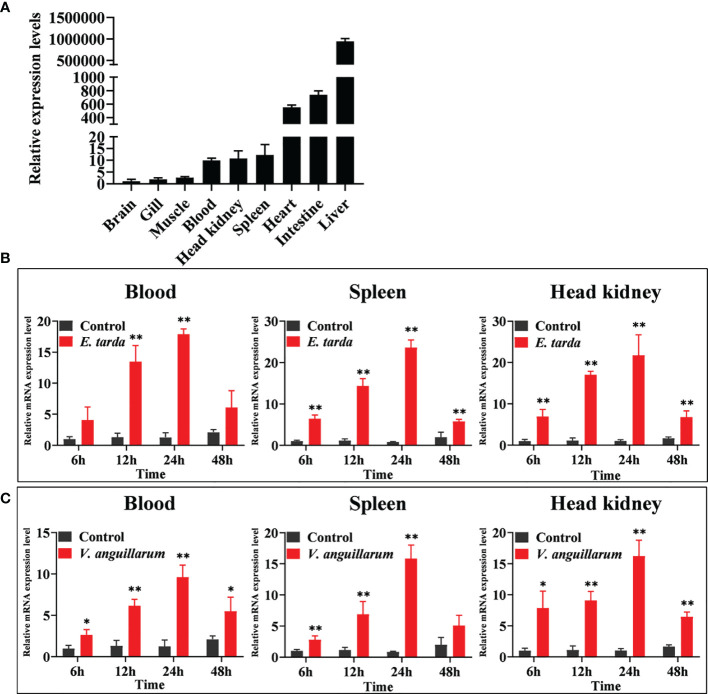
The expression of PoC3 in Japanese flounder tissues with and without bacterial infection. **(A)** PoC3 expression in the brain, gill, muscle, blood, head kidney, spleen, heart, intestine, and liver of Japanese flounder was determined by quantitative real time RT-PCR. For convenience of comparison, the expression level in brain was set as 1. **(B, C)** PoC3 expression after bacterial challenge. Japanese flounder were infected with or without (control) *Edwardsiella tarda* **(B)** or *Vibrio anguillarum* **(C)** and PoC3 expression in blood, spleen, and head kidney was determined by quantitative real time RT-PCR at various time points. In each case, the expression level of the control fish was set as 1. Data are shown as means ± SEM (N = 3). N, the number of times the experiment was performed. **P < 0.01; *P < 0.05.

qRT-PCR analysis showed that constitutive PoC3 expression occurred, in increasing order, in the brain, gill, muscle, blood, head kidney, spleen, heart, intestine, and liver of Japanese flounders, with the expression level in liver drastically higher than the expression of other tissues (**Figure 3A**). When the fish were infected with the bacterial pathogen *E. tarda*, significant inductions of PoC3 expression were detected in blood at 12 and 24 h post-infection (hpi), with the highest level of induction occurring at 24 hpi (17.9-fold) (**Figure 3B**). In spleen and head kidney, *E. tarda* infection induced PoC3 expression to significant levels at 6, 12, 24, and 48 hpi, with the highest expression level of induction occurring at 24 hpi (23.6- and 21.7-fold respectively) (**Figure 3B**). When the fish were challenged with *V. anguillarum*, PoC3 expression was significantly upregulated at 6, 12, 24, and 48 hpi in blood and head kidney, and the expression of PoC3 in spleen increased significantly at 6, 12, and 24 hpi (15.8-fold), and fell back to normal level at 48 hpi (**Figure 3C**).

### 3.3 Binding and Bactericidal Effect of PoC3 to Bacteria

**Figure 4:**
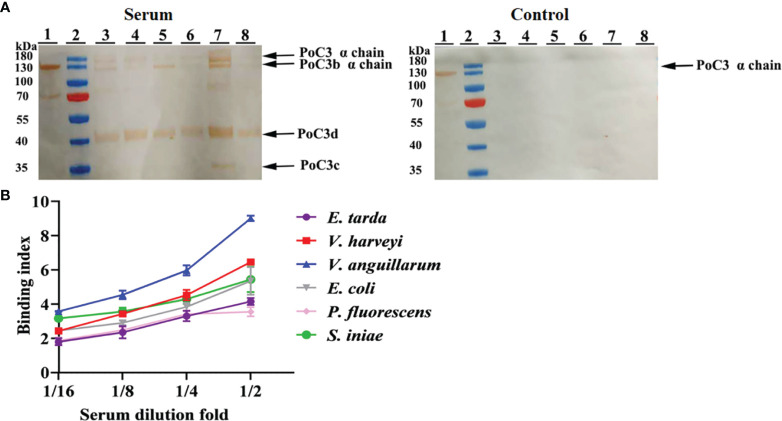
Serum PoC3 binding to bacteria. **(A)** Different bacteria were incubated with or without (control) serum for 1 h, and Western blot was performed to detect bacteria-bound serum C3 with mouse anti-PoC3 polyclonal antibody. Lane 1, Japanese flounder serum. Lane 2, protein marker. Lane 3 to 8 are *Vibrio harveyi, Edwardsiella tarda, Escherichia coli, Streptococcus iniae, Vibrio anguillarum* and *Pseudomonas fluorescens*. **(B)** Different bacteria were incubated with or without different dilutions fold Japanese flounder serum, and PoC3 in serum interacted with bacteria was determined by ELISA. Data are the means of three independent assays and presented as means ± SEM.

**Figure 5:**
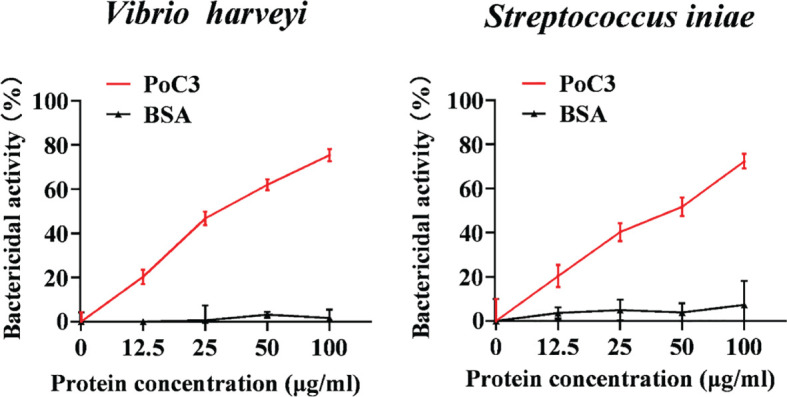
The bactericidal activity of PoC3. *Vibrio harveyi* and *Streptococcus iniae* were incubated with or without different concentrations of PoC3 or BSA for 1 h. The bacterial survival was determined by plate count. Data are shown as means ± SEM (N = 3). N, the number of times the experiment was performed.

In order to examine the binding effect of PoC3 to bacteria in the serum, the different bacteria were incubated with the serum of Japanese flounder or PBS (control). The western blot analyses with mouse anti-PoC3 polyclonal antibody (45) showed that PoC3 in the serum can bind to *V. harveyi*, *E. tarda*, *E. coli*, *S. iniae*, *V. anguillarum* and *P. fluorescens*, among which *V. anguillarum* had the strongest binding ability, but no binding phenomenon was found in the control group (**Figure 4A**). In addition, PoC3 could be degraded into activated fragments after incubation with bacteria in serum, including C3c and C3d. ELISA analysis showed that PoC3 in serum exhibited apparent binding to *E. tarda*, *E. coli*, *P. fluorescens*, *V. anguillarum*, *V. harveyi*, and *S. iniae* in a manner that depended on the concentration of serum (**Figure 4B**). To examine the bactericidal effect of the PoC3, different concentrations of PoC3 were incubated with *V. harveyi*, *S. iniae*, *E. tarda*, *E. coli*, *V. anguillarum* and *P. fluorescens*. The results showed that PoC3 exhibited a direct bactericidal activity to *V. harveyi* and *S. iniae* in a manner that depended on the dose of the protein (**Figure 5**), whereas it had no significant effect on the survival of *E. tarda*, *E. coli*, *V. anguillarum* and *P. fluorescens* (data not shown).

### 3.4 Involvement of PoC3 in Complement Activation and Bacterial Infection

**Figure 6:**
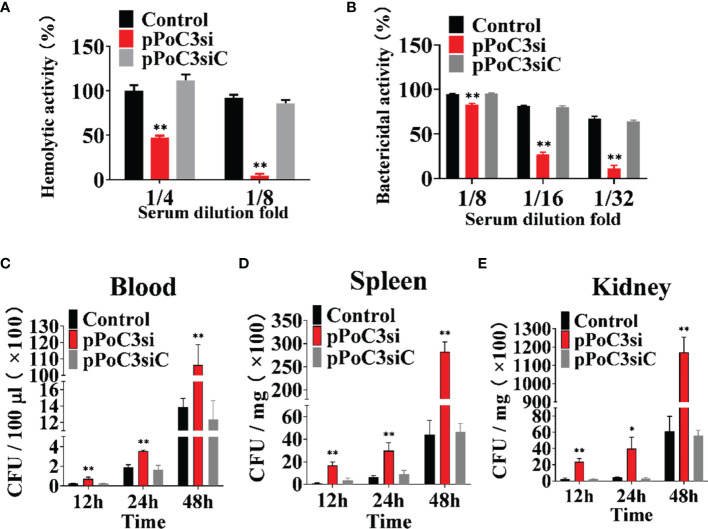
Effect of PoC3 knockdown on complement activation and bacterial infection. **(A, B)** Effect of PoC3 knockdown on hemolytic activity and bactericidal activity. Serum from Japanese flounder treated with pPoC3si, pPoC3siC, or PBS (control) was serially diluted and incubated with rabbit red blood cells **(A)** and *Escherichia coli* **(B)**, respectively. At 1 h after incubation, the hemolytic **(A)** and bactericidal **(B)** activities of the serum were determined. **(C–E)** Effect of PoC3 knockdown on bacterial infection. Japanese flounder pre-administered with or without (control) pPoC3si or pPoC3siC were infected with *Edwardsiella tarda*, and bacterial numbers in blood **(C)**, spleen **(D)** and kidney **(E)** were determined at various time points. Data are shown as means ± SEM (N = 3). N, the number of times the experiment was performed. *P < 0.05, **P < 0.01.

To examine the effect of PoC3 on complement activation, PoC3 expression in Japanese flounder was knocked down by RNA interference (RNAi). qRT-PCR analysis showed that in Japanese flounder administered with pPoC3si-2, which expresses a PoC3-targeting small interfering RNA (siRNA), the expression of PoC3 in blood, spleen, and kidney was most significantly inhibited compared to that in the control fish (**Figure S1**). Therefore, we used pPoC3si-2 for the knockdown experiment and renamed pPoC3si-2 as pPoC3si. After incubation with rabbit erythrocytes, the serum of the fish treated with pPoC3si exhibited significantly decreased hemolytic activities at 1/4 and 1/8 dilution fold and bactericidal activities at 1/8, 1/16, and 1/32 dilution fold, whereas the serum of the fish treated with pPoC3siC exhibited hemolytic and bactericidal activities similar to that of the serum from the control fish (**Figures 6A, B**). To examine the effect of PoC3 knockdown on bacterial infection, pPoC3si- and pPoC3siC-treated Japanese flounder were infected with *E. tarda*, and bacterial recoveries from blood, spleen and kidney were determined at 12, 24, and 48 hpi. The results showed that in all examined tissues pPoC3si-treatd fish exhibited significantly higher bacterial recoveries than the control fish at all examined time points (**Figures 6C–E**). No apparent difference in bacterial recovery between pPoC3siC-treated fish and control fish was observed.

### 3.5 Binding of rPoC3a to Bacteria

**Figure 7:**
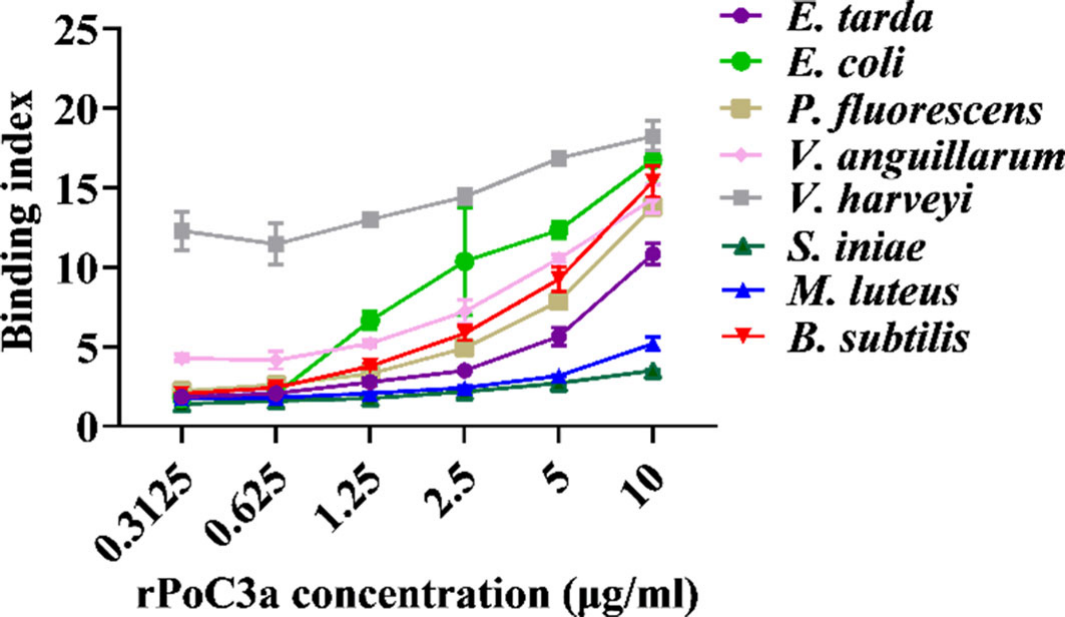
Binding of rPoC3a to bacteria. *Edwardsiella tarda, Escherichia coli, Pseudomonas fluorescens, Vibrio anguillarum, Vibrio harveyi, Streptococcus iniae, Micrococcus luteus and Bacillus subtilis* were incubated with or without different concentrations of rPoC3a, and bacteria-protein binding was determined by ELISA. Data are the means of three independent assays and presented as means ± SEM.

To examine the protein interact with bacteria, rPoC3a and rSumo were purified from *E. coli* as a Flag- or His-tagged protein (**Figure S2**), and the Gram-negative bacteria *E. tarda*, *E. coli*, *P. fluorescens*, *V. anguillarum*, and *V. harveyi*, and the Gram-positive bacteria *S. iniae*, *M. luteus* and *B. subtilis* were incubated with rPoC3a and rSumo in different concentrations. ELISA analysis showed that rPoC3a exhibited apparent binding to *E. tarda*, *E. coli*, *P. fluorescens*, *V. anguillarum*, *V. harveyi*, *S. iniae*, *M. luteus* and *B. subtilis* in a manner that depended on the dose of the protein (**Figure 7**). However, rPoC3a had no apparent bactericidal activity on *E. tarda*, *E. coli*, *P. fluorescens*, *V. anguillarum*, *V. harveyi*, *S. iniae*, *M. luteus* and *B. subtilis* (data not shown).

### 3.6 Effect of rPoC3a on Peripheral Blood Leukocytes (PBL)

**Figure 8:**
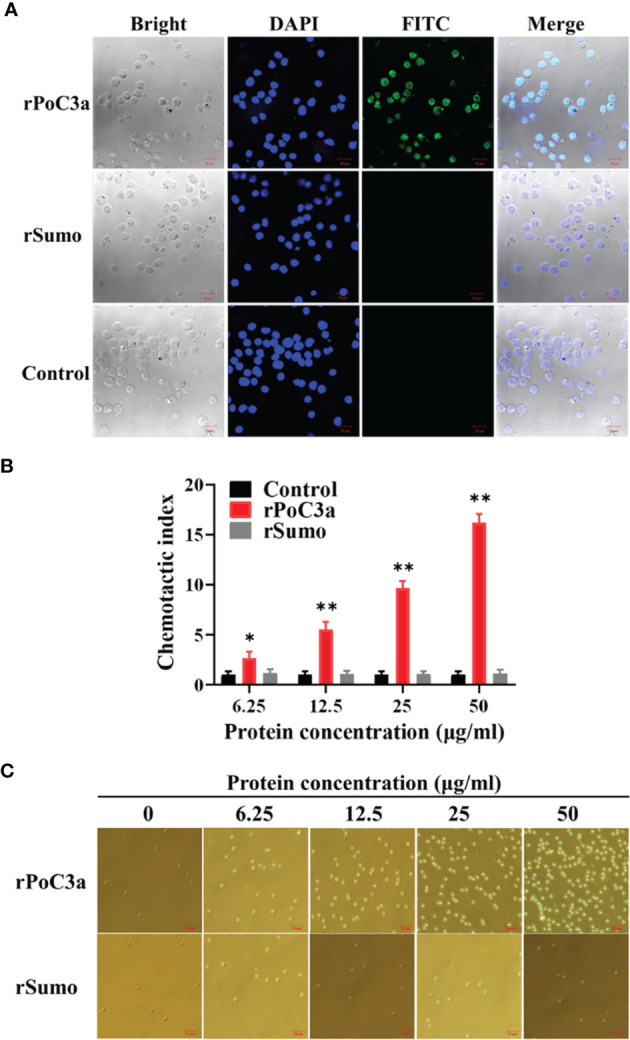
Binding of rPoC3a to peripheral blood leukocytes (PBL). **(A)** PBL was incubated with or without (control) rPoC3a or rSumo, and FITC-labeled goat anti-mouse IgG was used to detect the proteins bound to the cells. The cells were stained with DAPI and observed with a confocal microscope. Bar, 10μm. **(B, C)** Chemotactic activity of rPoC3a. The chemotactic activity of rPoC3a in various concentrations against PBL was determined using transwell migration assay. Chemotactic index was presented as fold increase in the number of migrated cells induced by rPoC3a compared to that induced by PBS (control). Data are shown as means ± SEM (N = 3). N, the number of times the experiment was performed. **P < 0.01; *P < 0.05. **(C)** The migrated cells induced by the rPoC3a in **(B)** were observed under a microscope. Bar, 20μm.

Immunofluorescence microscopy showed that following incubation of PBL with rPoC3, the protein was detected on the PBL cells (**Figure 8A**). In contrast, when PBL were incubated similarly with rSumo, no binding of the protein was detected on the PBL cells (**Figure 8A**). To further examine the effect of rPoC3, the chemotactic activity of rPoC3a was determined. The results showed that rPoC3a could induce the migration of PBL in a dose-dependent manner (**Figure 8B**). At the concentrations of 6.25, 12.5, 25, and 50 μg/ml, the numbers of migrated cells induced by rPoC3a were 2.6-, 5.5-, 9.6-, and 16.2-fold higher, respectively, compared to the control. When rPoC3a was added to both the upper and lower chambers of the transwell, cell migration was significantly inhibited, which suggested that migration was mediated by rPoC3a-induced chemotaxis rather than by chemokinesis (data not show). Consistently, microscopy results showed that rPoC3a induced migration of PBL in a manner that depended on the dose of the protein (**Figure 8C**).

### 3.7 Effect of rPoC3a Against Bacterial Infections

**Figure 9:**
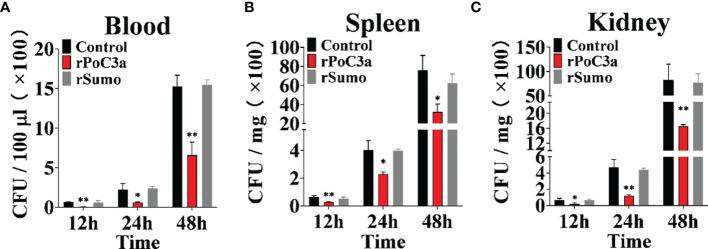
Effects of rPoC3a on bacterial infection. Japanese flounder pre-administered with or without (control) rPoC3a or rSumo were infected with *Edwardsiella tarda*, and bacterial numbers in blood **(A)**, spleen **(B)** and kidney **(C)** were determined at various time points. Data are shown as means ± SEM (N = 3). N, the number of times the experiment was performed. *P < 0.05, **P < 0.01.

To examine the effect of rPoC3a on pathogen infection, Japanese flounder were infected with *E. tarda* in the presence of rPoC3a, rSumo or PBS (control), and bacterial dissemination into blood, kidney, and spleen was subsequently determined at 12, 24, and 48 hpi. The results showed that at 12, 24, and 48 hpi, the amounts of *E. tarda* recovered from the three tissues in the fish infected with *E. tarda* plus rPoC3a were significantly lower than those from the control fish (**Figure 9**). For bacterial infections, the presence of rSumo had no apparent effect on pathogen burdens in the infected fish (**Figure 9**).

## 4 Discussion

In this study, we analyzed the sequence and expression of Japanese flounder C3, and examined biological effects of PoC3 and its activation fragment PoC3a. In mammals, the C3 is composed of two polypeptide chains α and ß, and contains macroglobulin domain, anaphylatoxin domain, thioester-containing domain and C345C domain (9, 55). Previous studies reported that domains of C3 have a variety of biological functions and play an important role in the process of immunity (56–58). In our study, sequence analysis showed that PoC3 is also composed of two polypeptide chains and possesses six domains: A2M_N_2, A2M, A2M_recep, Thiol-ester_cl, ANATO and C345C. These results suggested that PoC3 were basically consistent with higher vertebrates and even mammalian C3 in terms of structure and domains. The GCGEQ sequence is a highly conserved region in C3 of all species, and responsible for C3 to covalently link to the target molecule (10). In this study, we found that PoC3 contained the GCGEQ sequence, and a C3 convertase cleavage site was predicted in the LAR site. Sequence analysis revealed that PoC3 share high sequence identities with teleost C3. Furthermore, phylogenetic analysis showed that teleost C3 formed a clade that was distinctly separated from that formed by other vertebrate C3. These observations suggest that PoC3 may play an evolutionarily conserved role essential to the biological function of C3.

In mammals, C3 is mainly synthesized in liver cells (59). In southern catfish, *in situ* hybridization and RT-PCR indicated that C3 was highly expressed in the liver (27). In the grouper, mRNA expression level of C3 in liver was drastically higher than the other tissues (32). In Atlantic salmon, common carp, and wolfish, C3 also had high levels of expression in liver (29, 30, 60). In our study, the PoC3 was detected ubiquitously in all examined tissues of healthy fish, with relatively higher levels occurring in liver, intestine and heart, and low levels occurring in brain, which was similar to the C3 expression profiles in other teleost. Pathogen infection will increase the expression level of complement components (8, 27). In dojo loach, the expression level of C3 was significantly upregulated by *Aeromonas hydrophila* challenge in liver, spleen, skin, and gill (61). The mRNA expression level of C3 was induced after LPS stimulation in rainbow trout (62). Similar to these reports, in this study, the expression of PoC3 was induced to significant extents in blood, spleen and head kidney by *E. tarda* or *V. harweyi*. These results suggested that PoC3 was involved in host immune response against bacterial infection.

In mammals, C3 played an important role in immunity involved in opsonization and cytolysis of pathogens (63). Activation of the complement pathway results in cleavage of C3, and release of the anaphylatoxin C3a and deposition of C3b on the bacterial surface (64). After complement activation, C3b is cleaved to iC3b by factor I in the presence of the cofactor, and formation C3 final degradation products C3c and C3d (65). Previous studies found that serum C3 can bind to several pathogens, including *Legionella pneumophila* (66), *Chlamydia trachomatis* (67), *Leishmania major* (68), *Leishmania mexicana* (69), *Trypanosoma cruzi* (70), *Mycobacterium leprae* (71) and *Mycobacterium tuberculosis* (72). In this study, we investigated the binding effect of serum PoC3 to bacteria, and these results indicated that PoC3 can bind to several Gram-negative and Gram-positive bacteria in complement activation process. However, in previous report, there is no conclusion that C3 can directly kill bacteria. In teleost, several complement components can bind and inhibit the growth of bacteria (73, 74). In our study, we found that PoC3 can have a direct bactericidal activity to *V. harveyi* and *S. iniae*. These results indicated that C3 of teleost may have multiple strategies to eliminate pathogens and enhance innate immunity. Based on these results, we speculated that C3 interaction with bacteria played an important role in the elimination of bacterial infection.

In murine model, C3-deficient mice lack of antibody responded to T-cell-dependent antigen, resulting in impaired host immune response (12, 13). C3-deficient mice infected by *Streptococcus pneumoniae* exhibited significantly infection in the lungs and bloodstream, leading to an overwhelming inflammatory response and decreased survival times (75). Previous studies have shown that C3-deficient mice was significantly reduced in the ability of removing *E. coli* than wild-type mice, and the lost function in C3-deficient mice was restored by complementation with C3 protein (76). In lamprey, C3 knockdown decreased the survival rate of larvae against the infection with *A. hydrophila* (77). In pervious study, we found that recombinant trypsin-like serine protease domain of Fcator I inhibited complement activation by degrading C3b in the serum of Japanese flounder (45). Based on the previous results, in this study, we found that in Japanese flounder administered with pPoC3si, the mRNA level of PoC3 was significantly decreased, suggesting that PoC3 expression was successfully interfered by the small RNA expressed from the plasmid. In subsequent experiments, we found that pPoC3si treated fish serum exhibited significantly lower hemolytic and bactericidal activities than untreated normal serum or pPoC3siC-treated fish serum. Following exposure to *E. tarda* infection, fish with PoC3 knockdown displayed significantly increased number of *E. tarda* than the control fish. These results are consistent with the decreased hemolytic and bactericidal activities in pPoC3si-treated fish and suggest that PoC3 plays a positive role in the anti-infection immunity of Japanese flounder.

C3a has been demonstrated to possess a direct and potent antimicrobial effect against both Gram-negative and Gram-positive bacteria (25). In *B. japonicum*, recombinant BjC3a could inhibit the growth of bacteria in a dose-dependent manner (78). In mammals, C3a bound to the cell surface of bacteria, and induced bacterial membrane rupture (25). C3a-derived peptide also could bind to bacteria membrane (24). We found that rPoC3a exhibited binding activities to all eight examined bacterial species from Gram-positive and Gram-negative bacteria, the results indicating that rPoC3a have a wide range of bacterial targets. The binding indexes differed for different bacteria, suggesting that rPoC3a had different binding affinities for different types of bacteria. In addition, we also found that rPoC3a could not affect the survival and growth of bacteria. These results suggested that interaction of rPoC3a with target bacteria probably played a role in anti-bacterial immunity. In mammals, C3a has been shown to induce chemotaxis for eosinophils and mast cells, and promote the inflammation in immune response (36). In teleost, previous study has proved that recombinant C3a stimulated chemotaxis of macrophages (36). In mammals, C3a performed biological activity via binding to C3a receptors (C3aR), and C3a–C3aR interactions initiate chemotaxis *via* a C3aR-independent mechanism (79). In rainbow trout, C3a can bind to head kidney cells and cause respiratory burst (78). We also found that rPoC3a could bind to Japanese flounder PBL, and induce the migration of PBL. These results indicate that, as observed in higher vertebrates, rPoC3a may have a pro-inflammatory effect in the immune response of Japanese flounder by enhancing the activity of immune cells. Previous studies have shown that various immune molecules could bind to different glycoconjugates on bacterial cell surfaces (80). In mammals, C3a can bind to C3aR on the cell membrane (17, 79). We speculate that rPoC3a may bind to C3aR on Japanese flounder PBL. So rPoC3a likely binds to PBL and bacteria via different mechanisms. In mice, injection the C3a-derived peptide into mice significantly reduced the infection of *Streptococcus pyogenes* in the spleen (25). In mammals, C3a can interact with C3aR in the membrane of phagocytes and mast cells, inducing the cell migration and antibacterial effect (81, 82). In agreement with these observations, our *in vivo* study showed that in the presence of rPoC3a, *E. tarda* dissemination and colonization in tissues of Japanese flounder were significantly reduced, suggesting that rPoC3a activated host immune responses, which resulted in enhanced bacterial clearance.

In conclusion, the results of this study showed that PoC3 is a structural homologue of C3 and a key factor involved in complement activation. We demonstrated for the first time that PoC3 had direct bactericidal effect on certain bacteria. In addition, PoC3a plays a prominent role in inducing chemotaxis and antibacterial infection, which supports a role for PoC3a in antibacterial immunity. These observations provide new insights into the biological function of C3 and C3a in fish.

## Data Availability Statement

The original contributions presented in the study are included in the article/**Supplementary Material**. Further inquiries can be directed to the corresponding author.

## Ethics Statement

The animal study was reviewed and approved by Ethics Committee of Institute of Oceanology, Chinese Academy of Sciences.

## Author Contributions

M-fL and MW conceived the study. MW and B-bJ conducted the experiments and analyzed the data. MW wrote the manuscript. M-fL edited the manuscript. All authors contributed to the article and approved the submitted version.

## Funding

This work was supported by the grants from the National Natural Science Foundation of China (31972831), the National Key Research and Development Program of China (2018YFD0900501), and the Taishan Scholar Program of Shandong Province.

## Conflict of Interest

The authors declare that the research was conducted in the absence of any commercial or financial relationships that could be construed as a potential conflict of interest.

## Publisher’s Note

All claims expressed in this article are solely those of the authors and do not necessarily represent those of their affiliated organizations, or those of the publisher, the editors and the reviewers. Any product that may be evaluated in this article, or claim that may be made by its manufacturer, is not guaranteed or endorsed by the publisher.
