# Supplementary material for: Complement C3 and Activated Fragment C3a Are Involved in Complement Activation and Anti-Bacterial Immunity
Source: Front Immunol. 2022 Feb 25;13:813173. doi: 10.3389/fimmu.2022.813173 (PMC8913944; doi:10.3389/fimmu.2022.813173)

Supplementary data

Figure S1. Expression of *PoC3* in pPoC3si-administered fish. Japanese flounder were administered with pPoC3si-1、pPoC3si-2, pPoC3si-3 or PBS (control), and at 7 d post-plasmid administration, *PoC3* expression in blood, spleen, or kidney was determined by quantitative real time RT-PCR. In each case, the expression level of the control fish was set as 1. Values are shown as means ± SEM (N = 3). N, the number of times the experiment was performed. *P < 0.05, **P < 0.01.

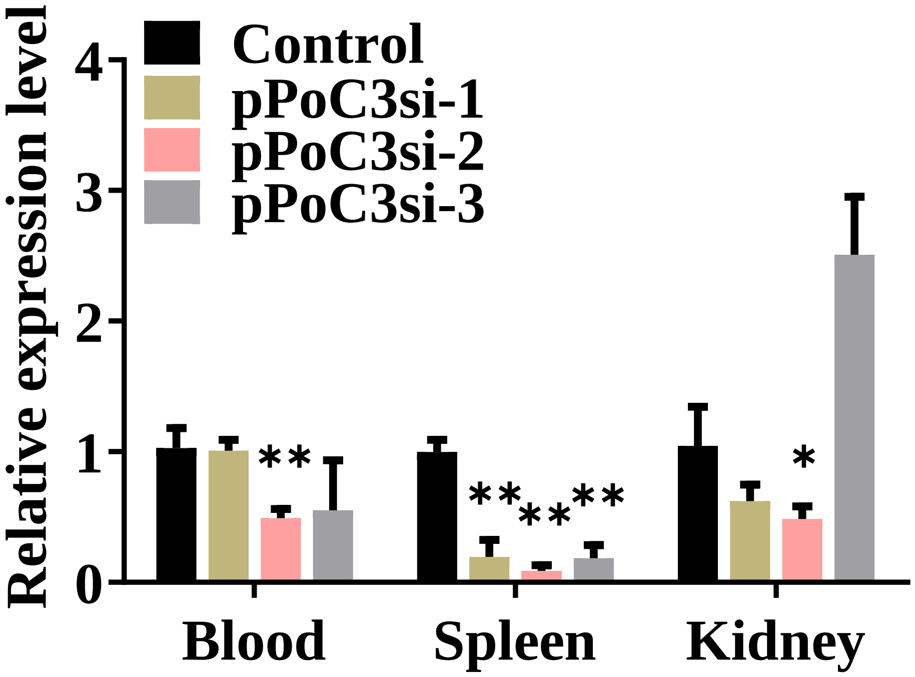

Figure S2. SDS-PAGE analysis of purified rPoC3a and rSumo. Purified rPoC3a and rSumo (lanes 2 and 3, respectively) were analyzed by SDS-PAGE and viewed after staining with Coomassie brilliant blue R-250. Lane 1, protein marker.

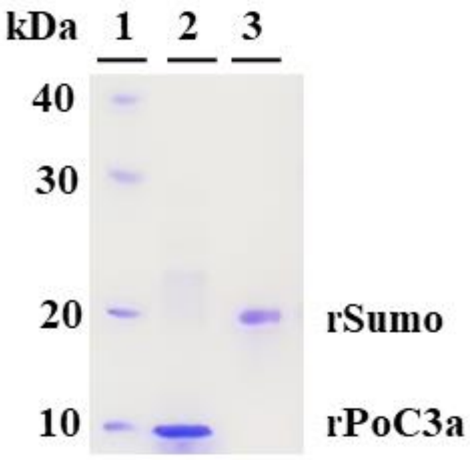

Supplement: Supplementary file 1 [file DataSheet_1.pdf]
